# Phylogenetic and Functional Analyses of Wheat *TaMAN* Genes Responding to Salinity and Pathogens

**DOI:** 10.3390/biology15171476

**Published:** 2026-09-01

**Authors:** Yanzhen Wang, Yanqi Wang, Jialu Li, Menglin Lei, Zhenchen Xie, Xia Liu

**Affiliations:** 1 Center for Agricultural Genetic Resources Research, Shanxi Agricultural University, Taiyuan 030031, China; wangyanzhen9605@163.com (Y.W.);; 2 Institute of Crop Sciences, College of Agriculture, Shanxi Agricultural University, Taigu 030801, China; 3 State Key Laboratory of Crop Gene Resources and Breeding, Institute of Crop Sciences, Chinese Academy of Agricultural Sciences, Beijing 100081, China

**Keywords:** wheat, endo-β-mannanases, *TaMAN* gene, salt stress, powdery mildew

## Abstract

Endo-β-1,4-mannanases (MANs) are glycoside hydrolase family 5 (GH5) enzymes that degrade cell wall mannan polysaccharides and participate in plant growth and stress adaptation. This gene family has not been systematically characterized in common wheat (*Triticum aestivum* L.). Here, we identified 24 *TaMAN* genes (*TaMAN1*–*TaMAN24*) genome-wide and analyzed their phylogeny, gene structures, chromosomal distribution, synteny, and promoter *cis*-acting elements. Expression profiles under biotic and abiotic stresses were investigated using public databases, salt-stress RNA-seq, and RT-qPCR. TaMAN proteins (386–475 aa) were mainly predicted to localize in the extracellular space. Phylogenetic analysis divided them into three groups, with Groups II and III representing monocot-specific expansions. Family expansion was driven primarily by whole-genome duplication, supplemented by tandem duplication on homoeologous group 6. Promoters were enriched in hormone- and stress-responsive *cis*-acting elements (ABRE, as-1/CGTCA-motif, W box). *TaMAN1*, *TaMAN5*, *TaMAN8*, *TaMAN9*, *TaMAN16* and *TaMAN19* were significantly induced by powdery mildew, while *TaMAN3*, *TaMAN4* and *TaMAN19*–*TaMAN22* rapidly responded to salt stress. This study provides candidate genes for disease-resistant and salt-tolerant wheat breeding.

## Simple Summary

Endo-β-1,4-mannanases (MANs) are cell wall-remodeling enzymes that regulate mannan polysaccharide turnover and plant stress adaptation. The *TaMAN* gene family in bread wheat (*Triticum aestivum* L.) has not yet been characterized. Here, we identified 24 *TaMAN* genes genome-wide and analyzed their evolutionary relationships, duplication patterns, protein properties, and *cis*-acting element distributions. Integrated expression profiling revealed distinct sets of *TaMAN* genes responsive to powdery mildew infection and early salt stress. This study provides a systematic understanding of the evolutionary and transcriptional features of wheat *TaMANs* and identifies candidate genes for breeding salt-tolerant and disease-resistant wheat.

## 1. Introduction

Common wheat (*Triticum aestivum* L., 2n = 6x = 42, AABBDD) is one of the most widely cultivated food crops worldwide, providing approximately 20% of the calories and protein consumed by humans. According to the Food and Agriculture Organization (FAO), global wheat production reached approximately 785 million tonnes in 2022, making it the second most produced cereal crop worldwide [1,2]. However, fungal diseases such as powdery mildew, stripe rust, leaf blotch and *Fusarium* head blight, together with abiotic stresses such as salinity and drought, have long threatened wheat production. Among them, powdery mildew, caused by *Blumeria graminis* f. sp. *tritici* (*Bgt*), is one of the most devastating diseases of wheat and can cause severe yield losses in epidemic years [3]. Breeding and growing resistant cultivars is the most economical and effective approach for disease control, the core of which lies in the mining of resistance gene resources and the dissection of resistance mechanisms. In recent years, the cloning of powdery mildew resistance genes such as *Pm21*, *TdCNL1*/*TdCNL5* and *Pm68*, the stripe rust resistance gene *Yr36* and the Fusarium head blight resistance gene *Fhb7* has greatly advanced wheat disease-resistance breeding [4,5,6]. Meanwhile, soil salinization is another major environmental constraint affecting global agricultural production, and dissecting the mechanisms of salt tolerance and mining salt-tolerance genes are equally important for the genetic improvement of wheat [7]. Notably, biotic and abiotic stresses often occur concomitantly in the field, and plant responses to the two types of stress overlap extensively at the level of signal transduction; therefore, simultaneously mining candidate genes for disease resistance and stress tolerance from the same gene family is of unique research value.

The plant cell wall serves as the first physical barrier against pathogen invasion, and its degradation products can act as damage-associated molecular patterns (DAMPs) to activate immune responses [8,9,10]. Many cell-wall proteins and plasma-membrane immune receptors are glycoproteins that require N-glycan processing in the endoplasmic reticulum (ER) and Golgi apparatus for proper folding and function [11,12,13]. Mannans—including linear mannan, glucomannan, galactomannan and galactoglucomannan—are hemicellulosic polysaccharides built on β-1,4-linked mannose backbones [14,15]. In addition to their structural roles, mannans function as storage carbohydrates mobilized during germination [14,16], and mannan-derived oligosaccharides can act as signaling molecules regulating wall biosynthesis and cell differentiation [17,18].

Endo-β-1,4-mannanase (MAN; EC 3.2.1.78) catalyzes the random hydrolysis of β-1,4-mannosidic linkages within the mannan backbone and thus occupies a central position in mannan turnover in plants [19,20]. In the sequence-based classification of glycoside hydrolases (GHs) deposited in the CAZy database [21,22], plant MANs belong predominantly to glycoside hydrolase family 5 (GH5), whose members adopt a (β/α)8 TIM-barrel fold and hydrolyze their substrates through a retaining double-displacement mechanism involving two catalytic glutamate residues [23]. Phylogenetic analyses have subdivided GH5 into more than 50 subfamilies, with plant mannanases clustering mainly in subfamily GH5_7 [24], and biochemical characterization of *Arabidopsis* thaliana MANs has confirmed their endo-acting activity on galactomannan and glucomannan substrates [25,26].

Plant MANs have been most extensively characterized in the context of seed germination, in which they weaken the mannan-rich micropylar endosperm of eudicot seeds, or the coleorhiza of grass caryopses, to permit radicle protrusion, and subsequently participate in the mobilization of endosperm reserves [27,28,29]. Multiple MAN isozymes have also been detected in the endosperm of cereal grains, including barley and wheat [30,31]. Beyond seeds, MANs contribute to fruit softening during tomato ripening [32,33] and to secondary wall remodeling during xylem differentiation in poplar, where PtrMAN6-derived oligosaccharides act as signals that suppress secondary wall thickening [17]. Notably, accumulating evidence implicates MANs in stress adaptation: *AtMAN3* promotes cadmium tolerance in *Arabidopsis* through the glutathione-dependent pathway [34]; heterologous expression of *MirMAN* from *Mirabilis jalapa* enhances salt tolerance, antioxidant capacity and root plasticity in *Arabidopsis* [35]; and a stress-induced GH5 glycosidase containing a fascin-like domain has been described in rice [36]. Because the cell wall is also the first structural barrier encountered by invading pathogens and a rich source of wall-derived danger signals [37,38,39], wall-remodeling enzymes such as MANs are plausible, yet still largely unexplored, components of crop stress responses.

The *MAN* gene family has been characterized at the genome-wide level in *Arabidopsis* [25], tomato and soybean [40], and *Brachypodium distachyon* [28]. These findings suggest that *MAN* family members are functionally diverse in plant stress adaptation, with distinct action directions and mechanisms among species and members. To date, however, no genome-wide systematic analysis of the *MAN* gene family has been reported in common wheat, and the expression characteristics and potential functions of this family under pathogen stress remain largely unknown. The availability of the fully annotated wheat reference genome [41], the transcriptional landscape of polyploid wheat [42] and public expression platforms such as expVIP [43] now renders such an analysis feasible.

In this study, we performed a genome-wide identification and systematic characterization of the *TaMAN* gene family in wheat, analyzing their physicochemical properties, conserved motifs, gene structures, phylogeny, chromosomal localization, synteny, and promoter *cis*-acting elements. We further investigated the expression patterns of *TaMAN* genes under biotic and abiotic stresses using public databases, salt-stress RNA-seq, RT-qPCR, and subcellular localization prediction. This work provides a foundation for understanding *MAN* gene functions in wheat stress adaptation and offers candidate gene resources for disease-resistant and salt-tolerant wheat breeding.

## 2. Materials and Methods

### 2.1. Identification and Physicochemical Property Analysis of TaMAN Family Members

The genome sequence, protein sequences and annotation files of common wheat (IWGSC RefSeq v1.1) were downloaded from the Ensembl Plants database (https://plants.ensembl.org/index.html, accessed on 2 March 2026). The hidden Markov model of the conserved GH5 endo-β-1,4-Mannanase domain (PF26410) was downloaded from the Pfam database (http://pfam.xfam.org/, accessed on 4 March 2026) [44], and HMMER 3.0 (E-value < 1 × 10^−5^) was used to search the wheat protein database [45]; meanwhile, BLASTP alignment (E-value < 1 × 10^−10^) was performed using the reported *Arabidopsis thaliana* MAN proteins as queries. After merging the two sets of results and removing redundancy, the integrity of the conserved domains of the candidate proteins was verified using the NCBI-CDD database (https://www.ncbi.nlm.nih.gov/Structure/bwrpsb/bwrpsb.cgi, accessed on 5 March 2026) [46], and *TaMAN* family members were finally identified and named according to their chromosomal positions. The amino acid number, molecular weight, theoretical isoelectric point, instability index, aliphatic index and grand average of hydropathicity (GRAVY) of the proteins were analyzed using the ExPASy ProtParam tool (https://web.expasy.org/protparam/, accessed on 8 March 2026) [47], and subcellular localization was predicted using the WoLF PSORT online tool (https://wolfpsort.hgc.jp/, accessed on 18 March 2026) [48] and DeepLoc 2.1 (https://services.healthtech.dtu.dk/services/DeepLoc-2.1/, accessed on 18 March 2026).

### 2.2. Phylogenetic, Conserved Motif and Gene Structure Analyses

MAN proteins from wheat, *Arabidopsis thaliana*, rice and maize were subjected to multiple sequence alignment using ClustalW, and a phylogenetic tree was constructed with MEGA 11 using the neighbor-joining (NJ) method with 1000 bootstrap replicates [49]; the tree was visualized using the iTOL online tool (https://itol.embl.de/upload.cgi, accessed on 2 April 2026) [50]. Conserved motifs of the TaMAN proteins were analyzed using the MEME online tool (https://meme-suite.org/meme/tools/meme, accessed on 3 April 2026), with the maximum number of motifs set to 10 and the motif width set to 6–50 aa [51]. Information on exons, introns and untranslated regions of the TaMAN genes was extracted from the genome annotation file (GFF3), and the motif and gene structure diagrams were drawn using TBtools II v2.487 [52].

### 2.3. Chromosomal Localization and Synteny Analyses

Genomic coordinates of all *TaMAN* family members were retrieved from the wheat reference genome GFF3 annotation file, and their chromosomal distribution map was visualized using the Gene Location Visualize from GTF/GFF module embedded in TBtools software. Syntenic relationships among the *TaMAN* genes within the wheat genome were analyzed using One Step MCScanX module (E-value < 1 × 10^−5^) TBtools II [53]. Genome data of *Arabidopsis thaliana*, rice, maize, *Aegilops tauschii*, *Triticum turgidum* and *Triticum dicoccoides* were downloaded from databases such as Ensembl Plants, and interspecific synteny maps between wheat and these species were constructed using MCScanX and visualized with TBtools II and Circos [54].

### 2.4. Analysis of Cis-Acting Elements in Promoters

The 2000 bp promoter sequences upstream of the start codons of the 24 *TaMAN* genes were extracted using TBtools and submitted to the PlantCARE database (https://bioinformatics.psb.ugent.be/webtools/plantcare/html/, accessed on 10 April 2026) for the prediction of *cis*-acting elements [55]. The identified elements were classified and statistically analyzed according to their functions, and the element distribution map was drawn using the Gene Structure View module in TBtools II v2.487.

### 2.5. Expression Analysis Under Stresses Based on Public Expression Databases

Transcriptome expression data (TPM) of *TaMAN* genes under cold (SRP064598), PEG-simulated drought (SRP068165), drought and heat combined stress (SRP045409), and infection by *Fusarium graminearum* (ERP013829), stripe rust (CYR31), powdery mildew (E09) (SRP041017) and *Zymoseptoria tritici* (ERP009837) were downloaded from the expVIP wheat expression database (https://www.wheat-expression.com/, accessed on 12 April 2026) [43]. After log2(TPM + 1) transformation, expression heatmaps were drawn using TBtools II v2.487 [52].

### 2.6. Salt Stress Treatment, Transcriptome Sequencing and Data Analysis

The salt-tolerant wheat cultivar Dekang 961 was used as the experimental material. Seedlings were hydroponically cultured with commercial wheat nutrient solution (Cat. NSP1070, Coolaber, Beijing, China) in a light incubator under a 16 h light/8 h dark photoperiod at 25 °C until reaching the two-leaf-one-heart stage. Subsequently, seedlings were divided into two groups: the salt treatment group was supplied with nutrient solution supplemented with 200 mM NaCl (S), while the control group (CK) was cultured in normal nutrient solution without additional NaCl. Leaf samples were collected at 6 h (S6), 12 h (S12) and 24 h (S24) after treatment, with the 6 h control (CK6) as the reference, and three biological replicates were set for each treatment. Total RNA was extracted using the TRIzol method, and cDNA libraries were constructed after quality inspection and sequenced on an Illumina platform with 150 bp paired-end reads. Raw data were filtered using fastp [56], aligned to the wheat reference genome using HISAT2 [57], and gene read counts were obtained using featureCounts [58]. Differential expression analysis was performed using DESeq2 with thresholds of padj < 0.05 and |log2FC| ≥ 1 [59]. Time-series expression pattern clustering of the DEGs was performed using the Mfuzz package with the cluster number set to 8 [60].

### 2.7. Material and Treatment Under Powdery Mildew Inoculation

The wheat landrace Aiganqianjing (AGQJ), collected in Shanxi Province, is a powdery mildew-resistant genotype, and was used as the plant material in this study. After a 10-min disinfection in 1% (*v*/*v*) sodium hypochlorite, seeds were washed several times with sterile distilled water and then allowed to germinate in the dark at 25 °C for 24 h. The resulting seedlings were transferred to a controlled-environment chamber maintained at 25 °C, with a 16 h/8 h (light/dark) cycle and a photon flux density of 300 μmol·m^−2^·s^−1^. When plants reached the two-leaf stage, conidia of *Blumeria graminis* f. sp. *tritici* isolate E09 were gently dusted onto the leaf surfaces. Leaf tissues were harvested before inoculation (0 hpi) and at 12, 24 and 48 h post-inoculation, snap-frozen immediately in liquid nitrogen, and kept at −80 °C until RNA isolation. Each sampling time point included three independent biological replicates.

### 2.8. RT-qPCR Analysis

To characterize how *TaMAN* genes respond transcriptionally to salt stress and powdery mildew (*Bgt*) infection, representative family members were subjected to reverse transcription quantitative PCR (RT-qPCR) analysis. Following the manufacturer’s instructions, total RNA was isolated with the UNIQ-10 Column Trizol Total RNA Isolation Kit (B51131), after which first-strand cDNA was prepared with the MightyScript Plus First Strand cDNA Synthesis Master Mix (gDNA digester) (B639252). Amplification reactions were set up with the 2× HyperMB Universal SYBR Green qPCR Master Mix (B690016) and run on a QuantStudio 6 Flex Real-Time PCR System (Thermo Fisher Scientific, Waltham, MA, USA), with the constitutively expressed *TaActin* gene serving as the reference for data normalization. Gene-specific primers were commercially synthesized by Shanghai Sangon Biotech (Shanghai, China). For every sampling point, three independent biological replicates were analyzed. Transcript abundance was quantified according to the 2^−ΔΔCT^ algorithm [61], and differences among treatments were evaluated by one-way ANOVA combined with Duncan’s multiple range test at *p* < 0.05 and *p* < 0.01, using GraphPad Prism (version 10.1.0).

## 3. Results

### 3.1. Identification and Basic Physicochemical Properties of TaMAN Members

Through the integrated analysis of BLASTP, HMMER, and NCBI-CDD validation, a total of 24 *TaMAN* genes, designated *TaMAN1*–*TaMAN24*, were identified from the Chinese Spring genome (IWGSC RefSeq v1.1). The physicochemical properties and predicted subcellular localizations of the encoded proteins are summarized in Supplementary Table S1. The TaMAN proteins ranged from 386 aa (TaMAN11) to 475 aa (TaMAN12) in length, with molecular weights of 43.21–52.34 kDa. The theoretical isoelectric points (pI) varied from 4.47 to 9.30: 13 members were acidic (pI < 7.0), whereas the remaining 11 members were neutral or alkaline (Table 1), implying that different TaMAN members may function in distinct pH microenvironments. The instability index ranged from 25.64 (TaMAN5) to 48.54 (TaMAN21), and the 13 members with an instability index below 40 were predicted to be stable proteins. The aliphatic index ranged from 67.14 to 88.63, and the grand average of hydropathicity (GRAVY) values were all negative (−0.288 to −0.143), indicating that all TaMAN proteins are hydrophilic. Subcellular localization prediction showed that the TaMAN proteins were mainly localized in the extracellular space, endoplasmic reticulum, lysosome/vacuole and Golgi apparatus. Among them, TaMAN1, TaMAN3, TaMAN5 and TaMAN10 were exclusively localized in the extracellular space, whereas TaMAN11 and TaMAN22 were predicted in the cytoplasm (TaMAN22 also in the extracellular space). These localization patterns are consistent with the biological roles of endo-β-1,4-mannanases in glycoprotein processing along the secretory pathway and in cell-wall polysaccharide metabolism.

### 3.2. Phylogenetic Analysis of the TaMAN Gene Family

**Figure 1:**
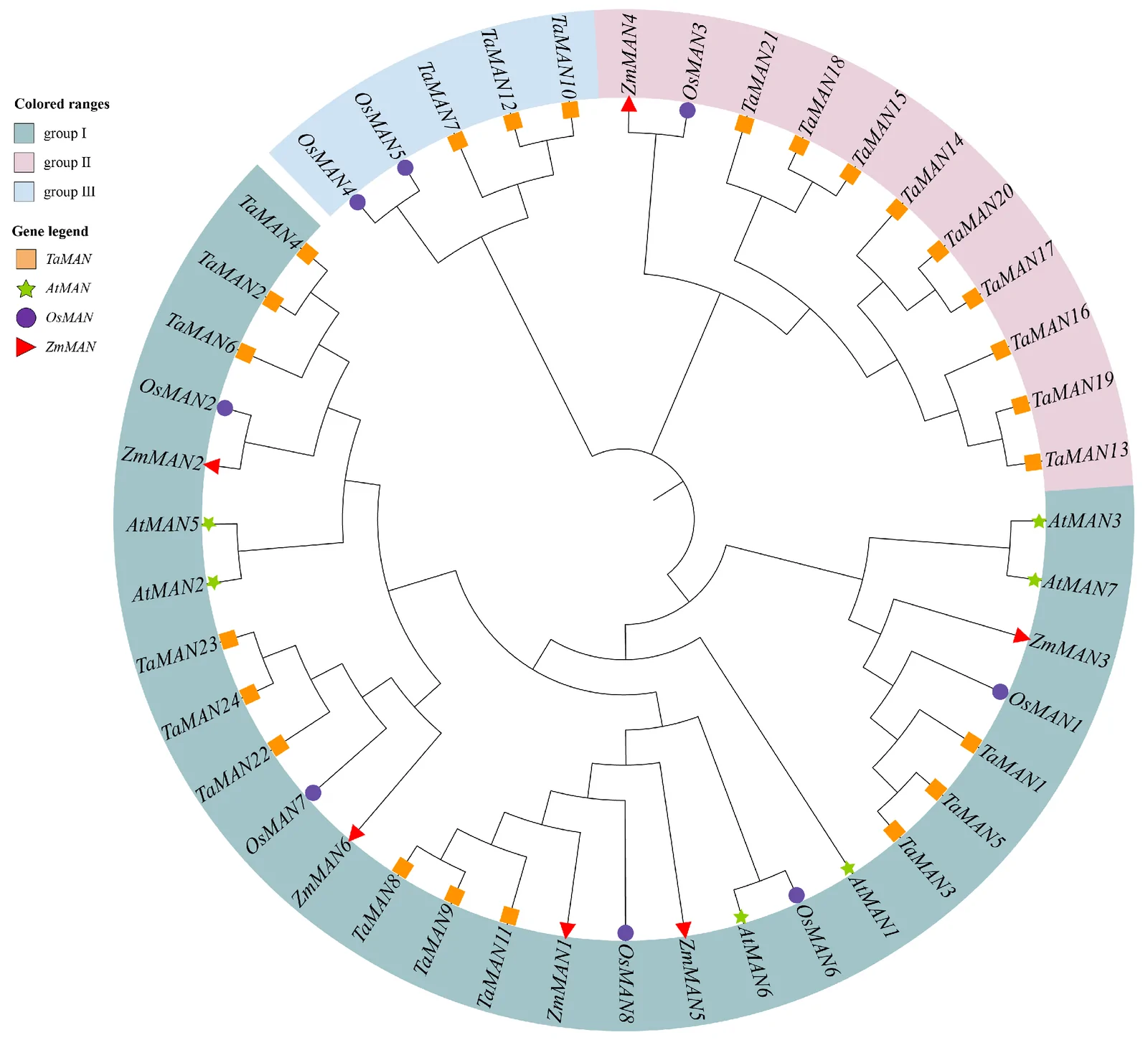
Phylogenetic tree of MAN proteins from wheat, *Arabidopsis*, rice and maize. Colored backgrounds indicate the three groups (Group I–III); orange squares, green stars, purple circles and red triangles represent MAN proteins from wheat (*TaMAN*), *Arabidopsis* (*AtMAN*), rice (*OsMAN*) and maize (*ZmMAN*), respectively.

To elucidate the evolutionary relationships of the *TaMAN* gene family, a phylogenetic tree was constructed using MAN proteins from wheat (*TaMAN*), *Arabidopsis thaliana* (*AtMAN*), rice *(OsMAN*) and maize (*ZmMAN*) (Figure 1, Table S2). All MAN proteins were divided into three groups (Group I–III). Group I was the largest clade, containing 12 *TaMAN* members (*TaMAN1*–*TaMAN6*, *TaMAN8*, *TaMAN9*, *TaMAN11* and *TaMAN22*–*TaMAN24*); Group II comprised nine *TaMAN* members (*TaMAN13*–*TaMAN21*); and Group III contained three members (*TaMAN7*, *TaMAN10* and *TaMAN12*). Notably, all *AtMAN* proteins clustered within Group I, whereas Groups II and III consisted exclusively of MAN proteins from monocots (wheat, rice and maize), suggesting a monocot-specific expansion of these two groups. Within each group, wheat MAN proteins were closely related to their rice and maize homologs, indicating that the *MAN* gene family is highly conserved among monocot species.

### 3.3. Conserved Motifs and Gene Structures of the TaMAN Gene Family

**Figure 2:**
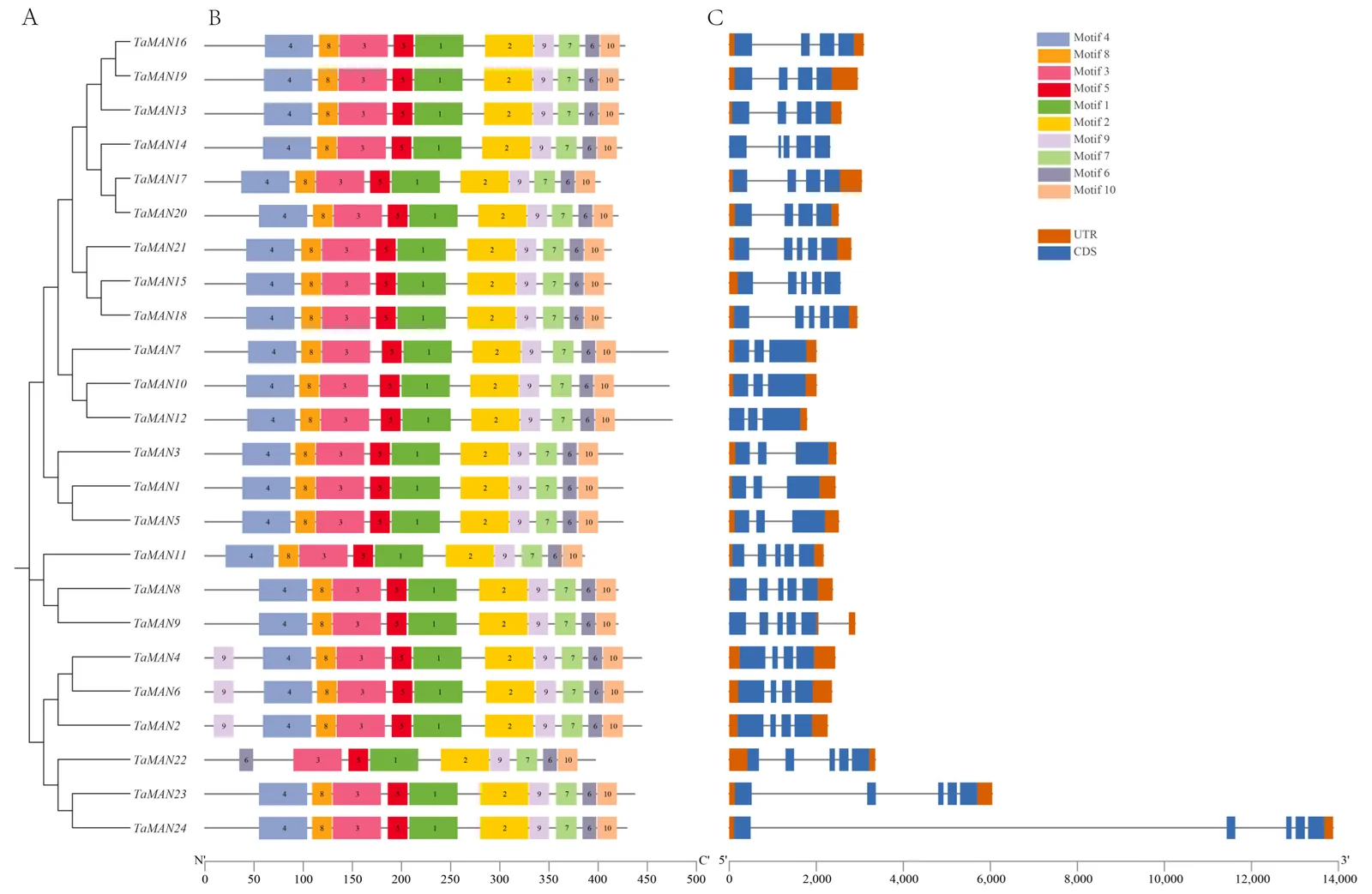
Phylogenetic relationships, conserved motifs and gene structures of the *TaMAN* family. (**A**) phylogenetic tree of TaMAN proteins; (**B**) distribution of conserved motifs, with Motifs 1–10 shown in different colors; (**C**) *TaMAN* gene structures, in which blue boxes represent coding sequences (CDS) and orange boxes represent untranslated regions (UTRs).

Conserved motif analysis of the 24 TaMAN proteins using the MEME suite identified a total of ten conserved motifs (Figure 2B). The vast majority of TaMAN proteins (22 out of 24) harbored all ten motifs, and the motifs were arranged in a highly consistent order (Motif 4-8-3-5-1-2-9-7-6-10), indicating that the primary structure of the TaMAN family is highly conserved. A few members exhibited motif variations: TaMAN22 lacked Motif 4 and Motif 8; TaMAN14 lacked Motif 5; and TaMAN2, TaMAN4 and TaMAN6 carried an additional Motif 9 at the N-terminus. Members with closer phylogenetic relationships shared similar motif compositions and arrangements, further corroborating the reliability of the phylogenetic analysis.

Gene structure analysis revealed that the coding sequences (CDS) of *TaMAN* genes consist of 3–5 exons, and members within the same clade exhibited similar exon–intron organizations (Figure 2C). Group II members (*TaMAN13*–*TaMAN21*) possessed compact gene structures, mostly with four exons, whereas the three Group III members (*TaMAN7*, *TaMAN10* and *TaMAN12*) each contained three exons. Notably, *TaMAN23* and *TaMAN24* harbored ultra-long introns, with genomic lengths of approximately 6 kb and 14 kb, respectively, markedly longer than those of the other members, suggesting that intron insertion events may have occurred during their evolution. In addition, most *TaMAN* genes contained 5′ and/or 3′ untranslated regions (UTRs). Intron phase analysis was further performed to examine the positional distribution of introns relative to codon boundaries. The results showed that phase 0 introns (located between codons) were the most prevalent in *TaMAN* genes, followed by phase 1 and phase 2 introns. The predominance of phase 0 introns is consistent with the evolutionary conservation of *GH5* gene structures and suggests that purifying selection has acted to maintain the reading frame of the catalytic domains during *TaMAN* family evolution.

### 3.4. Chromosomal Distribution of the TaMAN Genes

**Figure 3:**
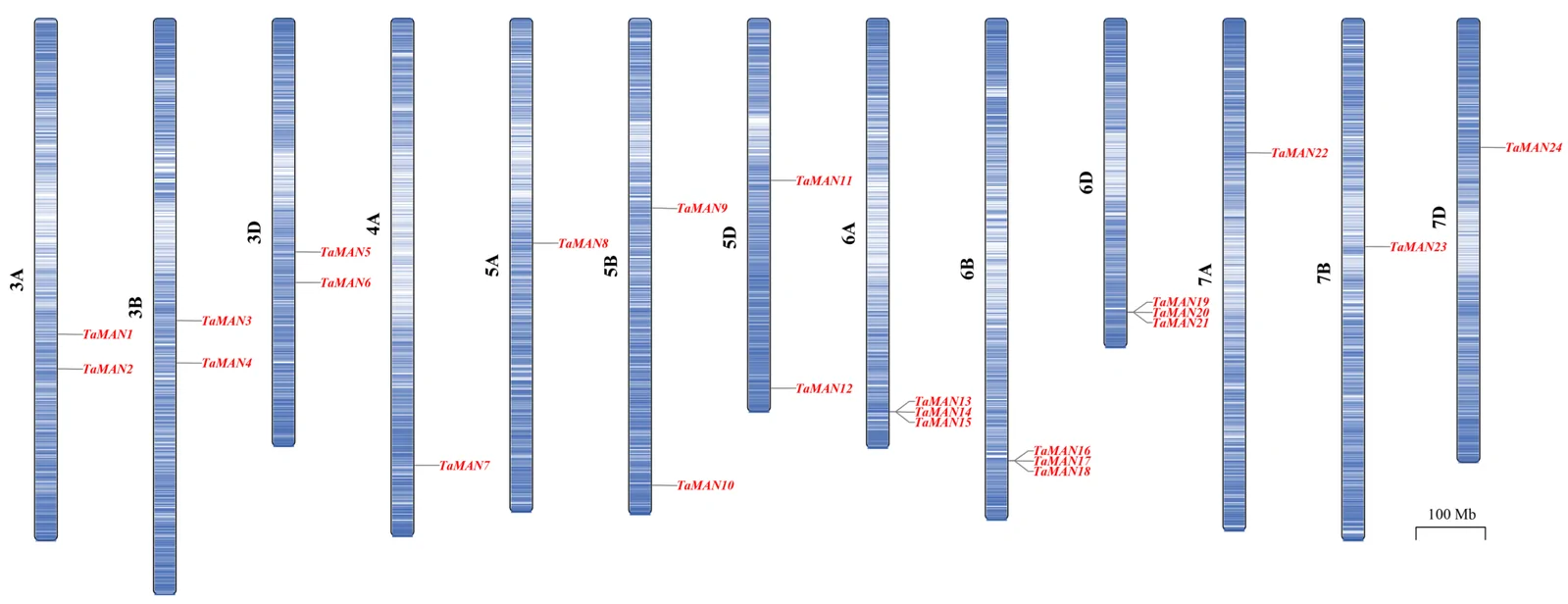
Chromosomal distribution of *TaMAN* genes in wheat. The scale bar represents the physical distance (Mb); gene names are shown in red.

Chromosomal localization analysis showed that the 24 *TaMAN* genes were unevenly distributed on 13 wheat chromosomes belonging to homoeologous groups 3, 4, 5, 6 and 7, whereas no *TaMAN* gene was detected on homoeologous groups 1 and 2 (Figure 3). Homoeologous group 6 harbored the largest number of *TaMAN* genes (nine in total, three on each of chromosomes 6A, 6B and 6D), where *TaMAN13*–*TaMAN15*, *TaMAN16*–*TaMAN18* and *TaMAN19*–*TaMAN21* formed tandem clusters on chromosomes 6A, 6B and 6D, respectively. Group 3 carried six genes (two on each of 3A, 3B and 3D), and group 5 carried five genes (one on 5A and two on each of 5B and 5D); chromosomes 4A, 7A, 7B and 7D each harbored a single *TaMAN* gene. At the subgenome level, the numbers of *TaMAN* genes were comparable among the A, B and D subgenomes (seven, eight and nine, respectively), and the genes on homoeologous groups 3, 5 and 6 displayed clear collinear distribution patterns among the three subgenomes. These results suggest that *TaMAN* genes were mainly retained and expanded through whole-genome duplication (polyploidization) during the evolution of hexaploid wheat, while the tandem arrays on group 6 indicate that tandem duplication events also contributed to the expansion of the family on this homoeologous group.

### 3.5. Intraspecific Synteny Analysis of the TaMAN Gene Family

**Figure 4:**
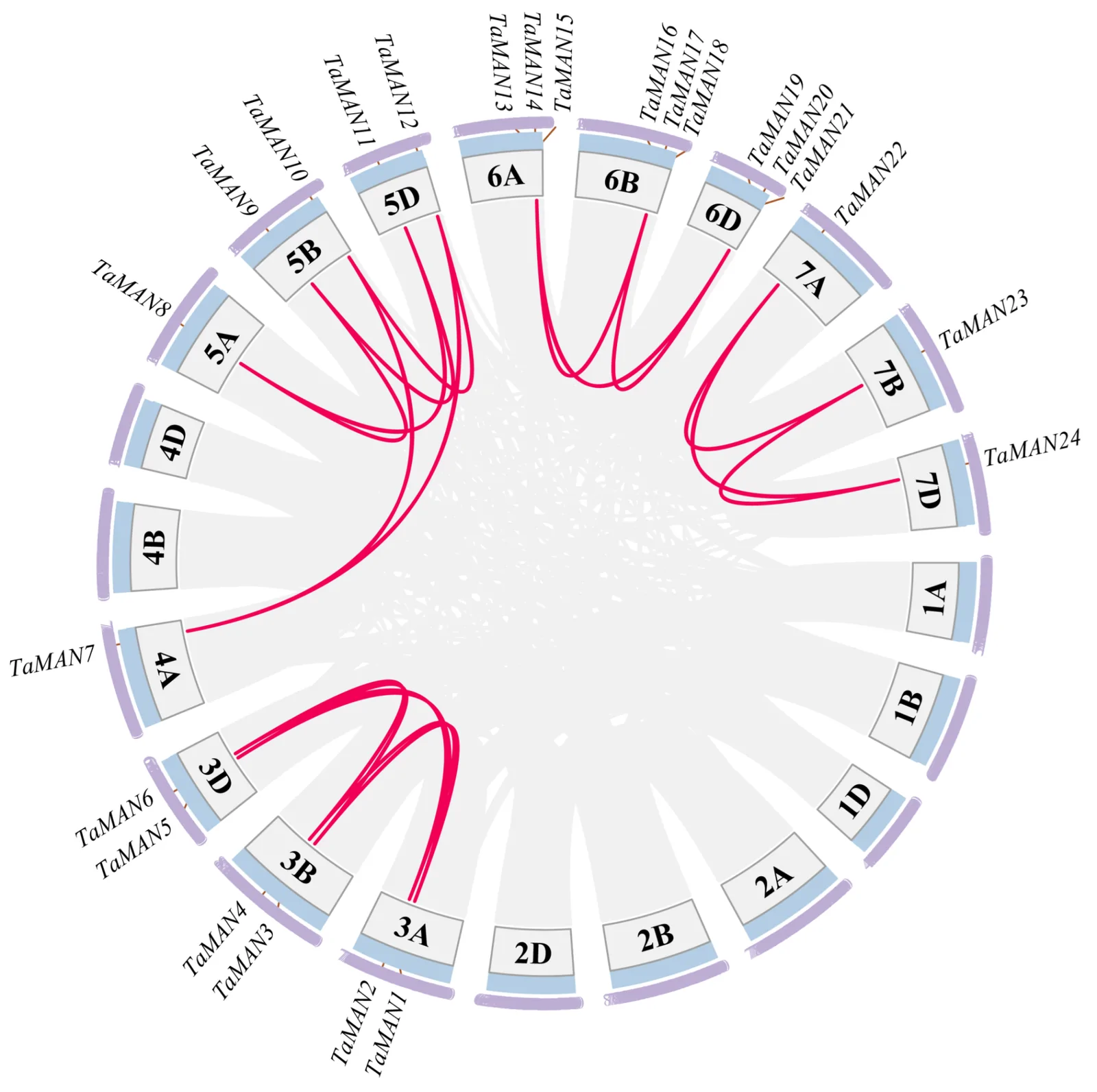
Intraspecific synteny analysis of *TaMAN* genes in wheat. Gray lines indicate syntenic blocks within the wheat genome, and red lines indicate syntenic relationships among *TaMAN* genes.

To investigate the expansion mechanism of the *TaMAN* gene family, intraspecific synteny analysis was performed among the *TaMAN* genes in the wheat genome (Figure 4). A large number of collinear gene pairs derived from segmental duplication were detected among the *TaMAN* genes, and these syntenic relationships were concentrated between the A, B and D subgenomes of homoeologous groups 3, 5, 6 and 7; that is, pairwise syntenic gene pairs were detected among 3A–3B–3D, 5A–5B–5D, 6A–6B–6D and 7A–7B–7D. In addition, *TaMAN7* on chromosome 4A was syntenic with *TaMAN10* (5B) and *TaMAN12* (5D), which may be associated with the chromosomal rearrangement (the 4AL/5AL translocation) that occurred during the evolution of wheat chromosome 4A. These results indicate that segmental duplication (whole-genome duplication/polyploidization) served as the major driving force for the expansion of the *TaMAN* gene family, and the one-to-one syntenic relationships among the A, B and D subgenomes within each homoeologous group further confirm the homoeology of the three subgenomes in hexaploid wheat. Together with the chromosomal localization results (Figure 3), the tandem clusters on homoeologous group 6 may suggest that tandem duplication also contributed to the local expansion of the family on chromosomes 6A, 6B and 6D.

### 3.6. Interspecific Synteny Analysis Between Wheat and Other Species

**Figure 5:**
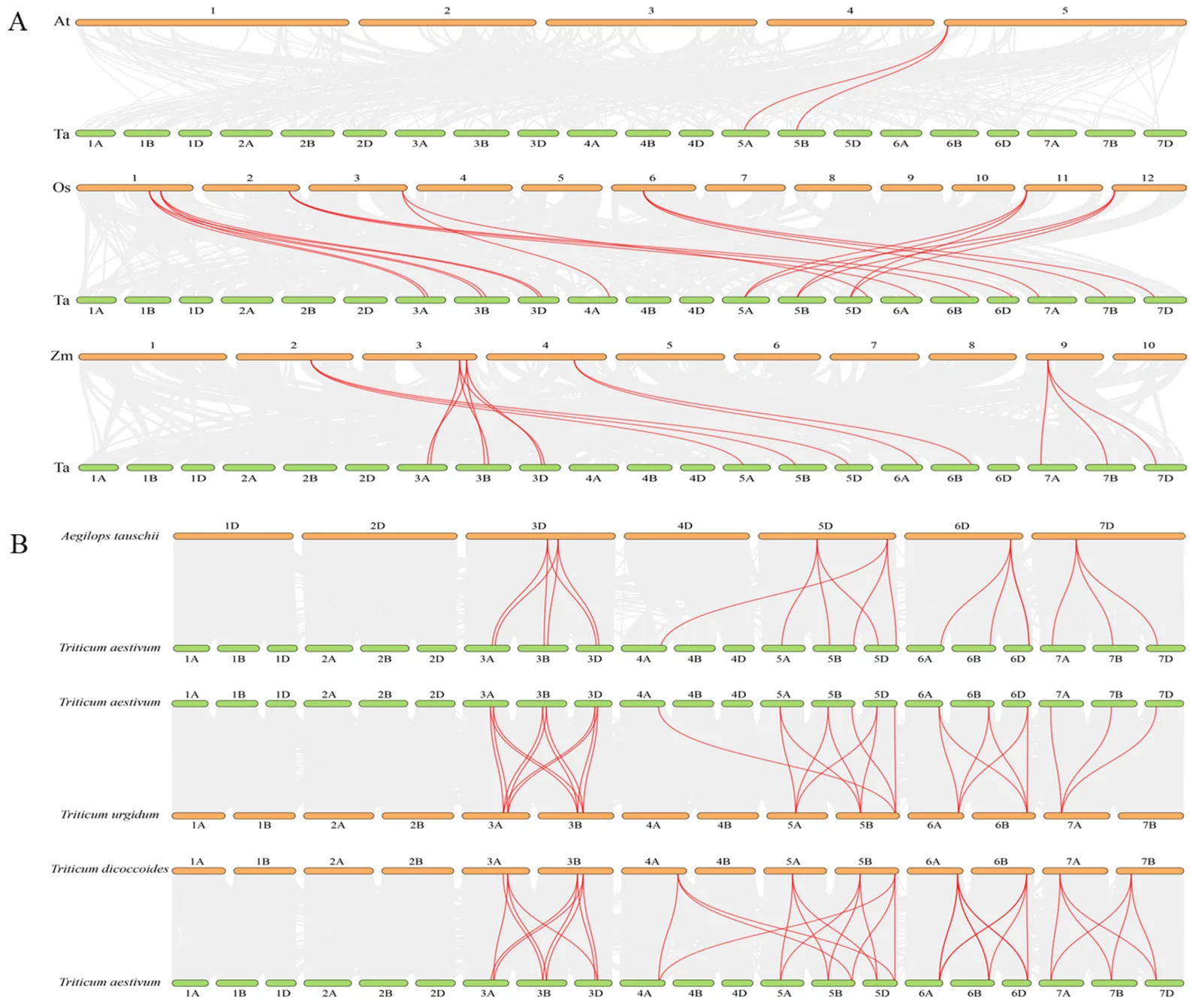
Interspecific synteny analysis of *MAN* genes between wheat and other species. (**A**) Synteny between wheat (Ta) and *Arabidopsis thaliana* (At), *Oryza sativa* (Os) and *Zea mays* (Zm); (**B**) synteny between wheat and *Aegilops tauschii*, *Triticum turgidum* and *Triticum dicoccoides*. Gray lines indicate interspecific syntenic blocks, and red lines indicate syntenic *MAN* gene pairs.

To further assess the evolutionary conservation of the *TaMAN* gene family and to trace the origin and retention of *TaMAN* genes during wheat evolution, interspecific synteny maps were constructed between wheat and *Arabidopsis thaliana*, rice and maize, as well as between wheat and its ancestral and related species (Figure 5, Table S3). Only two syntenic *MAN* gene pairs were identified between wheat and the dicot *Arabidopsis thaliana*, whereas 20 and 14 syntenic gene pairs were detected between wheat and the monocots rice and maize, respectively (Figure 5A), indicating that the synteny between wheat and monocots is much stronger than that with the dicot species. The syntenic genes were mainly distributed on the wheat chromosomes of homoeologous groups 3, 5, 6 and 7, as well as on rice chromosomes 1, 2, 3, 6, 11 and 12 and maize chromosomes 2, 3, 4 and 9, demonstrating that the *MAN* gene family is more evolutionarily conserved among monocots and that the *MAN* genes of wheat, rice and maize likely originated from common ancestral genes. Furthermore, 19, 32 and 38 syntenic *TaMAN* gene pairs were identified between wheat and its D-subgenome donor *Aegilops tauschii* (DD), the tetraploid relatives *Triticum turgidum* (cultivated emmer wheat, AABB) and *Triticum dicoccoides* (wild emmer wheat, AABB), respectively (Figure 5B), and the syntenic relationships were all concentrated on homoeologous groups 3, 5, 6 and 7. Notably, the *MAN* homologs of *Ae. tauschii* were syntenic not only with the D subgenome but also with the A and B subgenomes of wheat, reflecting the homoeology among the three subgenomes, whereas the *MAN* homologs of *T. turgidum* and *T. dicoccoides* (AABB) mainly corresponded to the members on the A and B subgenomes of wheat. These results indicate that *TaMAN* genes are highly conserved between wheat and its ancestral and related species, and were integrally retained without obvious loss of family members during wheat polyploidization and domestication.

### 3.7. Analysis of Cis-Acting Regulatory Elements in TaMAN Gene Promoters

**Figure 6:**
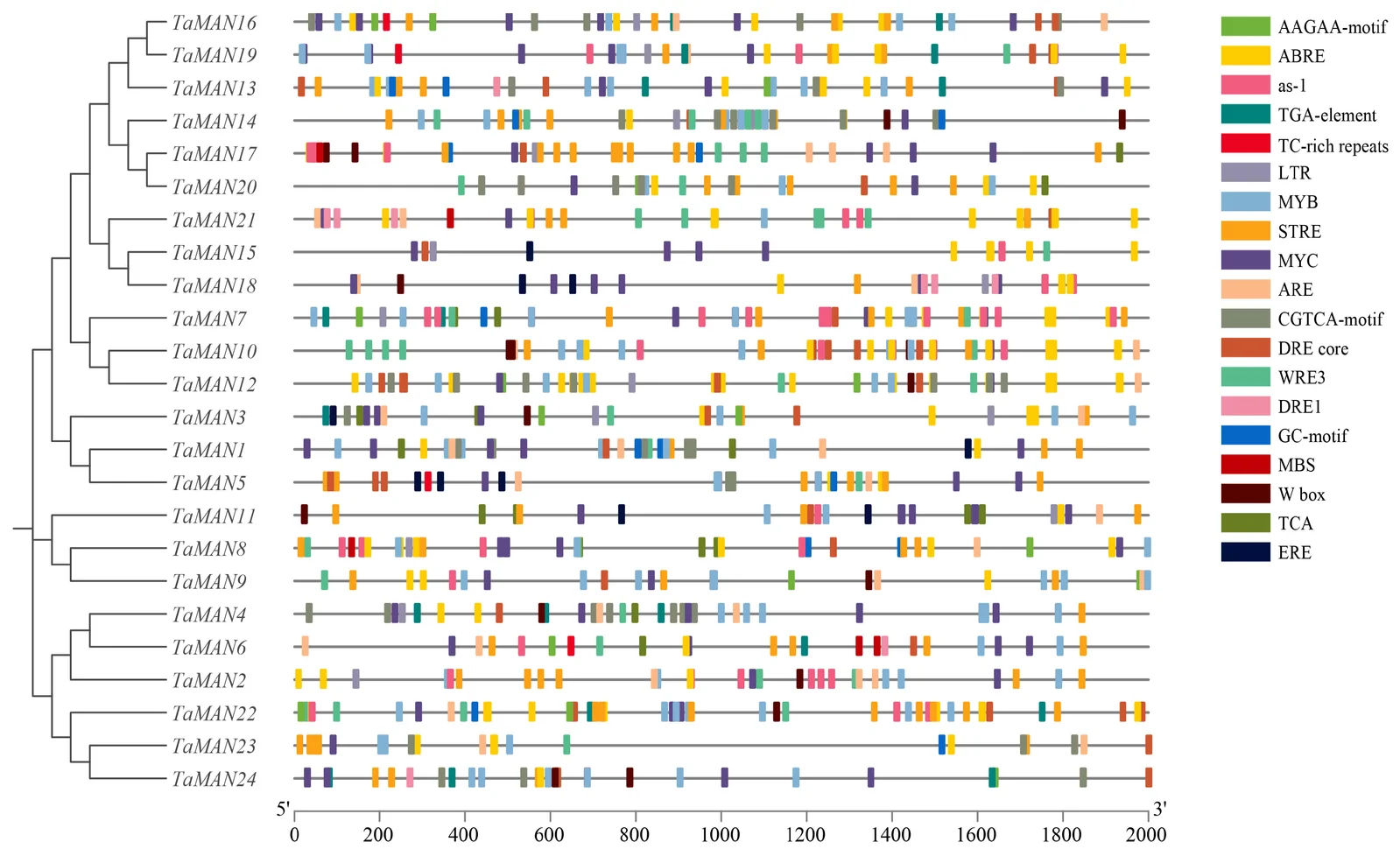
Distribution of *cis*-acting regulatory elements in the promoters of *TaMAN* genes. (**Left**): phylogenetic tree of TaMAN proteins; (**right**): distribution of *cis*-acting elements in the 2000 bp upstream promoter regions, with different colored boxes representing different element types.

To explore the potential transcriptional regulatory mechanisms of the *TaMAN* genes, *cis*-acting regulatory elements in the 2000 bp upstream promoter regions were predicted (Figure 6). A total of 849 *cis*-acting elements belonging to 19 types were identified in the promoters of the 24 *TaMAN* genes and were classified into four functional categories: hormone-responsive, abiotic stress-responsive, stress-responsive, and biotic stress- and immune-related elements (Table S4). Hormone-responsive elements were the most abundant (354, 41.7%), including the abscisic acid (ABA)-responsive element ABRE (122, the most abundant single element), the methyl jasmonate (MeJA)-responsive elements as-1 and CGTCA-motif (93 each), the auxin/salicylic acid-responsive TGA-element (18), the ethylene-responsive ERE (10), and the salicylic acid-responsive TCA and TCA-element (18 in total). Abiotic stress-responsive elements totaled 237 (27.9%), mainly comprising the stress-responsive element STRE (95), the drought/cold-responsive elements DRE core and DRE1 (49 in total), the anaerobic-responsive element ARE (34), the drought-responsive element MBS (14) and the low-temperature-responsive element LTR (15). MYB and MYC transcription factor binding sites numbered 112 and 83, respectively, and are broadly involved in stress signal transduction. Biotic stress- and immune-related elements totaled 63 (7.4%), including WRE3 (41), the pathogen-responsive and WRKY-binding W box (18) and the defense-related TC-rich repeats (4).

Notably, the promoters of all *TaMAN* genes contained ABRE, as-1/CGTCA-motif and MYC elements, indicating that hormonal (especially ABA and jasmonic acid) signaling and stress-responsive regulation are universal features of the *TaMAN* family. The number of elements per promoter ranged from 16 (*TaMAN15*) to 55 (*TaMAN7*), with an average of approximately 35; the Group III members (*TaMAN7*, *TaMAN10* and *TaMAN12*) harbored the largest total numbers of elements (49–55), and the *TaMAN12* promoter contained as many as 18 ABREs, the highest in the family, suggesting its potentially strong responsiveness to ABA signaling. These results indicate that *TaMAN* genes may be extensively involved in hormone signal transduction as well as biotic and abiotic stress responses, providing a theoretical basis for further investigation of the transcriptional regulation of *TaMAN* genes under pathogen stress.

### 3.8. Expression Patterns of TaMAN Genes Under Different Abiotic and Biotic Stresses

**Figure 7:**
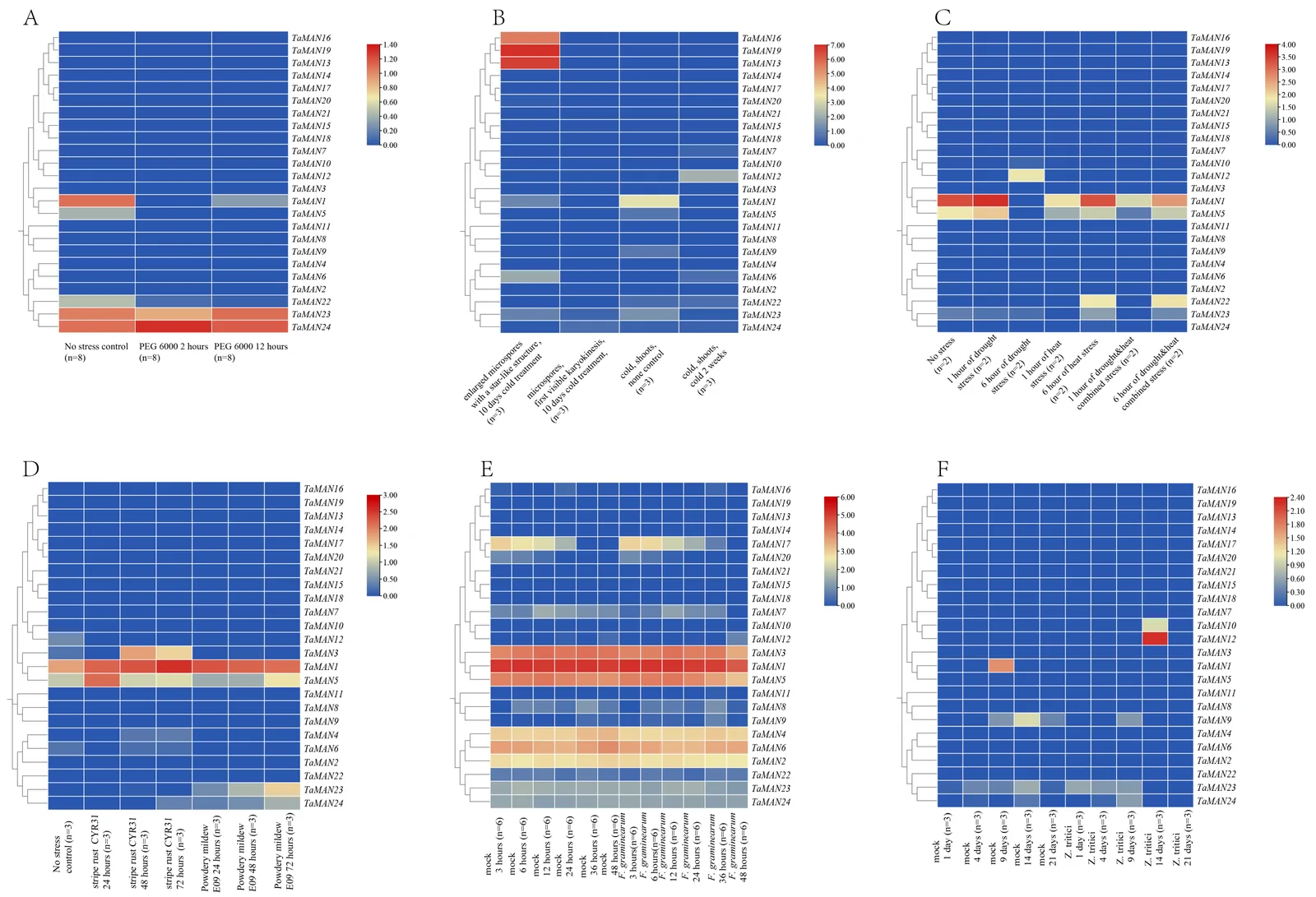
Expression heatmaps of *TaMAN* genes under different abiotic and biotic stresses. (**A**) PEG-simulated drought stress; (**B**) cold stress; (**C**) drought, heat and combined drought–heat stress; (**D**) stripe rust and powdery mildew inoculation; (**E**) *Fusarium graminearum* inoculation; (**F**) *Zymoseptoria tritici* inoculation. Colors from blue to red indicate expression levels from low to high.

To investigate the expression responses of *TaMAN* genes to abiotic stresses, the expression patterns of the 24 *TaMAN* genes under PEG-simulated drought, cold, drought and heat stress were analyzed using transcriptome data retrieved from public wheat expression databases (Figure 7A–C). Overall, most *TaMAN* genes were expressed at low or undetectable levels under these stresses, and only a subset of members exhibited marked stress- and tissue-specific responses, indicating pronounced functional divergence among the *TaMAN* family members. Under cold stress, the Group II members *TaMAN13*, *TaMAN16* and *TaMAN19* were specifically and highly expressed in enlarged microspores subjected to 10 days of cold treatment, but were barely expressed at other developmental stages or in shoots, whereas *TaMAN7* and *TaMAN12* showed increased expression in shoots after two weeks of cold treatment. Under PEG-simulated drought stress, *TaMAN23* and *TaMAN24* maintained relatively high expression levels at all time points, and *TaMAN24* was further up-regulated after 2 h of PEG treatment, showing a rapid response, whereas *TaMAN1*, which was highly expressed under control conditions, was down-regulated after PEG treatment. Under drought and heat stress, *TaMAN1* exhibited the most dynamic response: it was highly expressed under control conditions and after 1 h of drought, declined markedly at 6 h of drought, and was up-regulated again after 6 h of heat stress and combined drought-heat stress, displaying a typical dynamic response pattern; *TaMAN12* was induced after 1 h of heat stress, and *TaMAN22* showed elevated expression under the combined stress. Taken together, the responses of *TaMAN* genes to different abiotic stresses exhibited distinct member- and tissue-specific patterns, and members such as *TaMAN1*, *TaMAN23*, *TaMAN24* and *TaMAN12* responded to multiple stresses, representing important candidate genes of the *TaMAN* family involved in abiotic stress responses. These expression characteristics are consistent with the enrichment of stress-responsive *cis*-acting elements such as DRE, LTR and STRE in their promoters.

To further elucidate the expression responses of *TaMAN* genes to pathogen stress, the expression patterns of the 24 *TaMAN* genes under four biotic stresses, namely stripe rust (isolate CYR31), powdery mildew (isolate E09), *Fusarium graminearum* and *Zymoseptoria tritici*, were analyzed using transcriptome data retrieved from public wheat expression databases (Figure 7D–F). The responses of different *TaMAN* members to pathogen infection varied considerably. Following *F. graminearum* inoculation, *TaMAN1*, *TaMAN3*, *TaMAN5* and *TaMAN6* maintained high expression levels in both the mock controls and at all inoculated time points, showing constitutively high expression, whereas *TaMAN17* was relatively highly expressed at the early stage (3 h) after inoculation and declined thereafter. Under stripe rust infection, *TaMAN1* was continuously and highly expressed from 24 to 72 h post-inoculation (hpi), *TaMAN5* was strongly induced at 24 hpi and then declined, and *TaMAN3* was markedly up-regulated at 48–72 hpi; under powdery mildew infection, *TaMAN1* likewise maintained high expression, while *TaMAN5* and *TaMAN23* showed slight increases at the later stages. After *Z. tritici* inoculation, *TaMAN12* was strongly and specifically induced at 14 days post-inoculation (dpi), and *TaMAN10* was also up-regulated to some extent at the same time point, whereas the other members were barely expressed. Taken together, *TaMAN1* was highly expressed or induced under multiple pathogen stresses, representing the core candidate gene of the *TaMAN* family involved in pathogen stress responses; *TaMAN3* and *TaMAN5* mainly responded to rust and *Fusarium* infections, whereas *TaMAN12* specifically responded to *Z. tritici* infection, indicating pronounced functional divergence of the *TaMAN* family members in pathogen stress responses. These results are consistent with the enrichment of pathogen- and jasmonic acid-responsive *cis*-acting elements, such as W box and as-1/CGTCA-motif, in their promoters.

### 3.9. Principal Component Analysis of Wheat Transcriptome Samples Under Salt Stress

**Figure 8:**
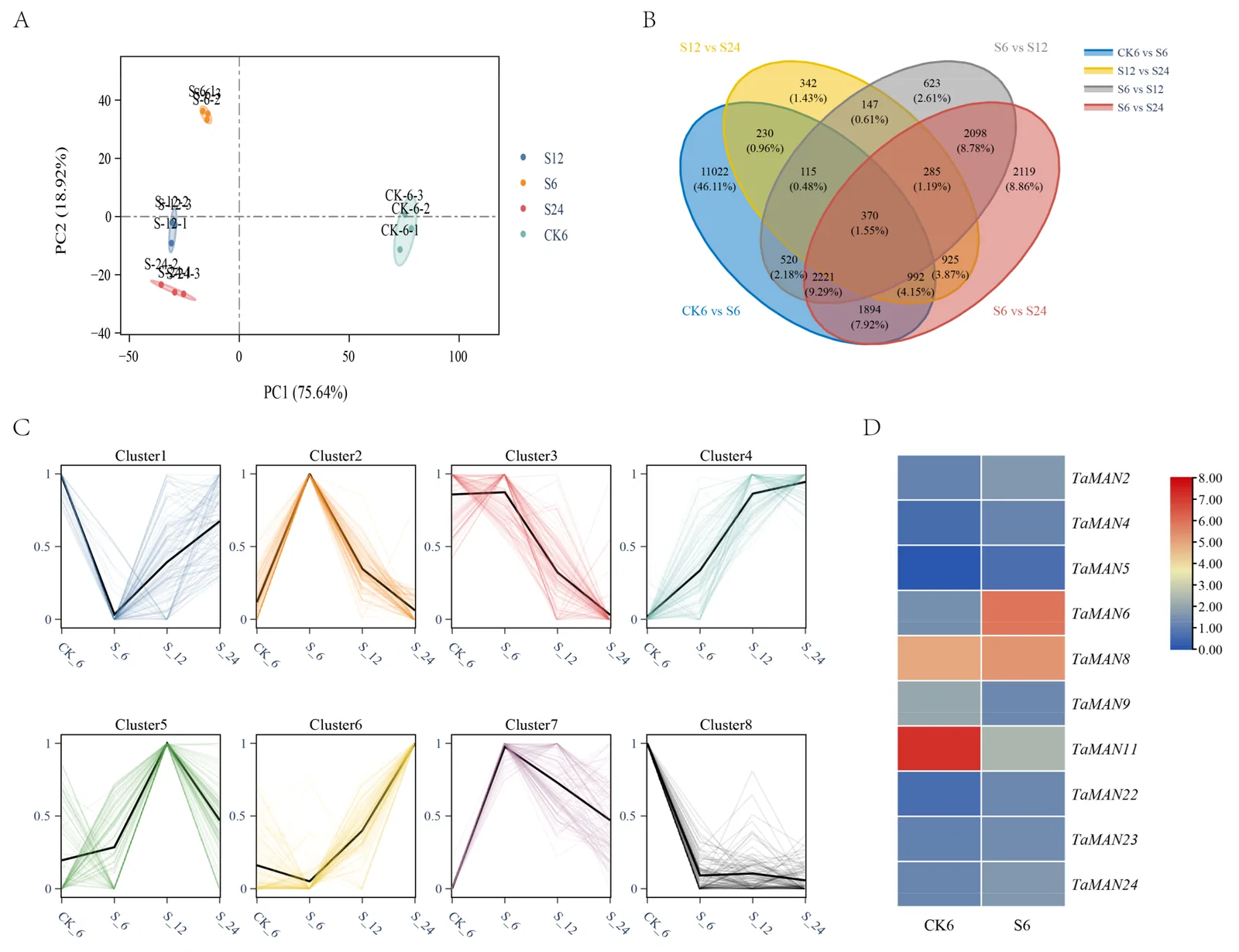
Transcriptome analysis of wheat under salt stress. (**A**) Principal component analysis (PCA) of the samples; (**B**) Venn diagram of DEGs among different comparisons; (**C**) time-series clustering of DEG expression patterns (Clusters 1–8); (**D**) expression heatmap of *TaMAN* genes in the CK6 and S6 samples.

Salt stress treatment was applied at the two-leaf-one-heart stage, and samples were collected from the control at 6 h (CK6) and from salt-treated plants at 6 h (S6), 12 h (S12) and 24 h (S24) for transcriptome sequencing. Principal component analysis (PCA) showed that the first and second principal components (PC1 and PC2) explained 75.64% and 18.92% of the variance, respectively, accounting for 94.56% cumulatively. The biological replicates of each group clustered tightly together, whereas the four sample groups were clearly separated: CK6 was distinctly separated from the salt-treated groups along PC1, and S6, S12 and S24 were distributed in an orderly manner along PC2 according to the treatment duration (Figure 8A). These results indicate that the transcriptome sequencing data exhibit good reproducibility and high reliability, that salt stress induced substantial transcriptome-level changes in wheat, and that the transcriptomic profiles differed markedly among treatment durations, making the data suitable for subsequent differential expression analysis.

### 3.10. Analysis of Differentially Expressed Genes in Wheat Under Salt Stress

Differentially expressed genes (DEGs) were screened using the thresholds of padj < 0.05 and |log2FC| ≥ 1. A total of 17,364 DEGs were identified in CK6 vs. S6, including 8933 up-regulated and 8431 down-regulated genes; 6379 DEGs were identified in S6 vs. S12 (3743 up-regulated and 2636 down-regulated); 10,904 DEGs in S6 vs. S24 (5121 up-regulated and 5783 down-regulated); and 3406 DEGs in S12 vs. S24 (1440 up-regulated and 1966 down-regulated) (Figure 8B). Venn diagram analysis showed that a total of 23,903 non-redundant DEGs were obtained across the four comparisons, of which 370 (1.55%) were shared by all four comparisons, constituting the core salt-responsive gene set, whereas 11,022 DEGs (46.11%) were specific to CK6 vs. S6, far exceeding the numbers of DEGs specific to the other comparisons. These results indicate that salt stress triggered large-scale transcriptional reprogramming in wheat as early as 6 h after treatment, and that the number of DEGs varied dynamically with treatment duration, with a large number of DEGs still present at 24 h compared with 6 h, suggesting that the transcriptional response of wheat to salt stress is a continuous and dynamically regulated process.

### 3.11. Time-Series Expression Pattern Analysis of Differentially Expressed Genes Under Salt Stress

To dissect the dynamic expression patterns of wheat in response to salt stress, time-series clustering analysis was performed on the DEGs across the salt stress time course, and the genes were divided into eight expression pattern clusters (Clusters 1–8) (Figure 8C, Table S5). Genes in Clusters 4 and 6 were continuously up-regulated with prolonged treatment, showing a sustained induction pattern; genes in Clusters 2 and 7 reached their expression peaks as early as 6 h after salt treatment, showing an early rapid-response pattern, after which Cluster 2 declined rapidly, whereas Cluster 7 remained highly expressed at 12 h; genes in Cluster 5 peaked at 12 h, showing a mid-stage response pattern; genes in Clusters 3 and 8 were continuously down-regulated after salt stress, showing a sustained repression pattern; and genes in Cluster 1 were markedly down-regulated at the early stage (6 h) and then gradually recovered, displaying a V-shaped expression pattern. These results demonstrate that the transcriptional response of wheat to salt stress exhibits distinct temporal specificity: early-responsive genes (Clusters 2 and 7) may be mainly involved in the perception of salt stress signals and rapid defense reactions, mid-stage-responsive genes (Cluster 5) in the amplification and transition of the stress response, and continuously induced genes (Clusters 4 and 6) in salt stress adaptation and homeostasis reconstruction, providing a basis for screening key salt-responsive genes at different stages.

### 3.12. Expression Analysis of TaMAN Genes at the Early Stage of Salt Stress

To clarify the expression responses of *TaMAN* genes at the early stage of salt stress, the expression levels (FPKM) of the *TaMAN* genes were extracted from the Dekang 961 transcriptome data and compared between the CK6 and S6 samples (Figure 8D, Table S6). Among the 24 *TaMAN* genes, ten were detectably expressed in the leaf transcriptome, namely *TaMAN2*, *TaMAN4*, *TaMAN5*, *TaMAN6*, *TaMAN8*, *TaMAN9*, *TaMAN11*, *TaMAN22*, *TaMAN23* and *TaMAN24*. Among them, *TaMAN11* showed the highest expression in the control (CK6) and decreased markedly to a low level after 6 h of salt treatment, indicating repression by salt stress; *TaMAN8* maintained relatively high expression under both control and salt-treated conditions, showing a constitutively high expression pattern; *TaMAN6* was markedly up-regulated after 6 h of salt treatment, showing induction by salt stress; *TaMAN9* was down-regulated after salt treatment; and *TaMAN2*, *TaMAN4*, *TaMAN5*, *TaMAN22*, *TaMAN23* and *TaMAN24* were expressed at low levels with little change. These results indicate that *TaMAN* family members exhibit diverse response patterns, including up-regulation, down-regulation and constitutive expression, at the early stage of salt stress, providing direct transcriptome-level evidence for the screening of salt-responsive candidate members.

### 3.13. RT-qPCR Validation of TaMAN Gene Expression Under Salt Stress

**Figure 9:**
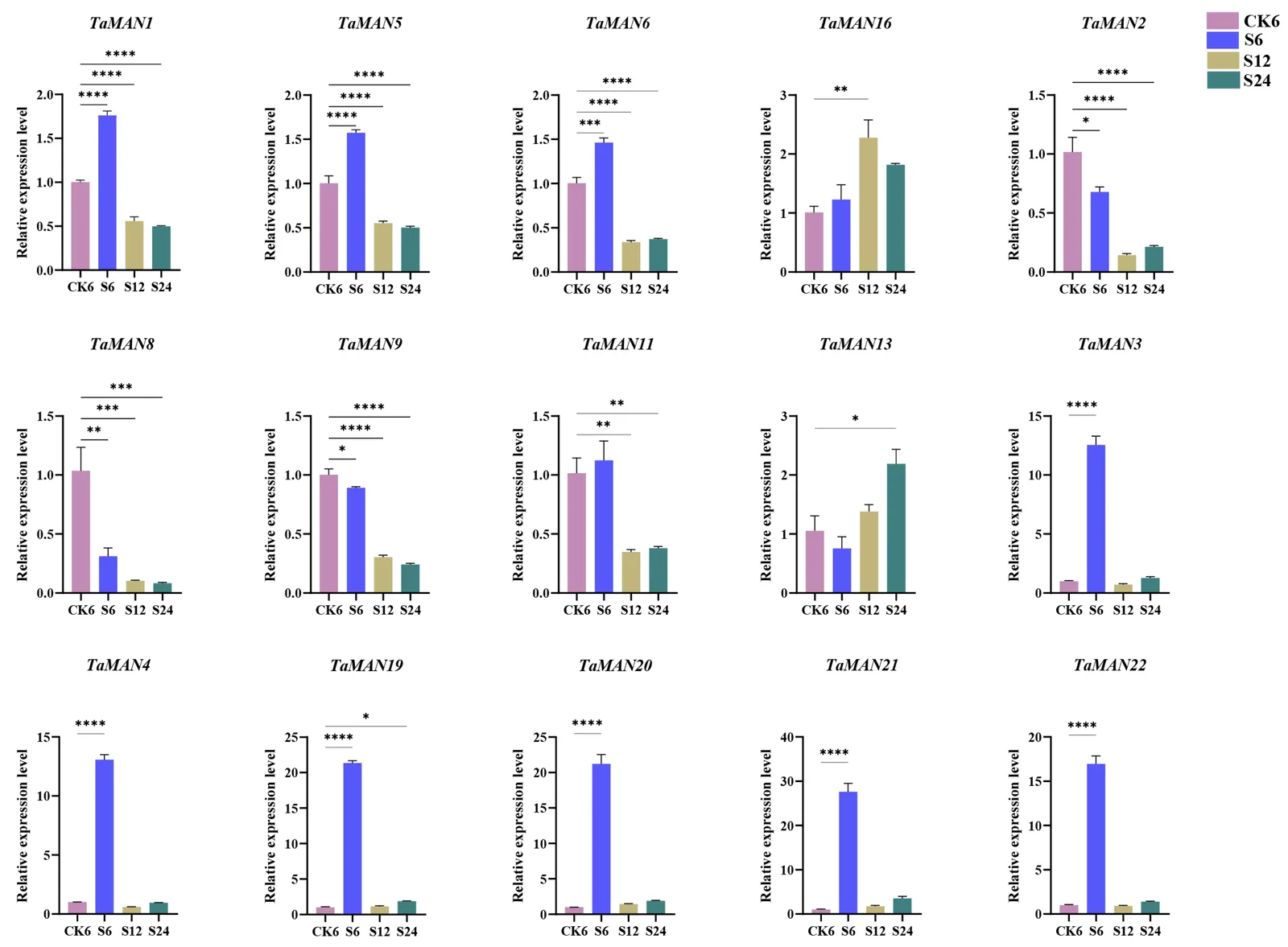
Differential expression of *TaMAN* genes in response to salt stress. *, **, ***, and **** indicate the significant correlations at the levels of *p* < 0.05, *p* < 0.01, *p* < 0.001, and *p* < 0.0001 respectively.

To verify the reliability of the salt-stress transcriptome data and to clarify the expression patterns of *TaMAN* family members under salt stress, fifteen *TaMAN* members were chosen for RT-qPCR profiling across the CK6, S6, S12 and S24 samples, aiming both to validate the salt-stress RNA-seq data and to resolve the salt-responsive behavior of the family (Figure 9, Table S7). A majority of the tested genes responded within the first 6 h of treatment: transcript levels of *TaMAN3*, *TaMAN4*, *TaMAN19*, *TaMAN20*, *TaMAN21* and *TaMAN22* rose steeply at S6 (12- to 28-fold relative to the control), after which they fell back progressively at S12 and S24, displaying a characteristic rapid early-response profile. *TaMAN1*, *TaMAN5* and *TaMAN6* followed the same temporal trend but with more moderate amplitudes, reaching maxima of roughly 1.5- to 1.8-fold at S6 before declining. In contrast, *TaMAN16* and *TaMAN13* accumulated gradually and attained their highest levels at S12 (~2.3-fold) and S24 (~2.2-fold), respectively, while *TaMAN2*, *TaMAN8* and *TaMAN9* were markedly repressed upon salt exposure. It is worth noting that, although the induction of several genes (e.g., *TaMAN5* and *TaMAN6*) agreed well between RNA-seq and RT-qPCR, members with low absolute transcript abundance in the sequencing data (e.g., *TaMAN22*) yield fold-change estimates that are sensitive to detection limits and are therefore best evaluated in conjunction with the qPCR measurements. Overall, the qPCR profiles closely mirrored the expression behaviors defined by the time-series clustering—rapid early induction in Clusters 2 and 7 and sustained up-regulation in Clusters 4 and 6—thereby corroborating the robustness of the transcriptome dataset. Collectively, *TaMAN3*, *TaMAN4*, *TaMAN19*, *TaMAN20*, *TaMAN21* and *TaMAN22* emerge as promising candidate genes mediating the wheat response to salt stress.

### 3.14. RT-qPCR Analysis of TaMAN Gene Expression Following Powdery Mildew Inoculation

**Figure 10:**
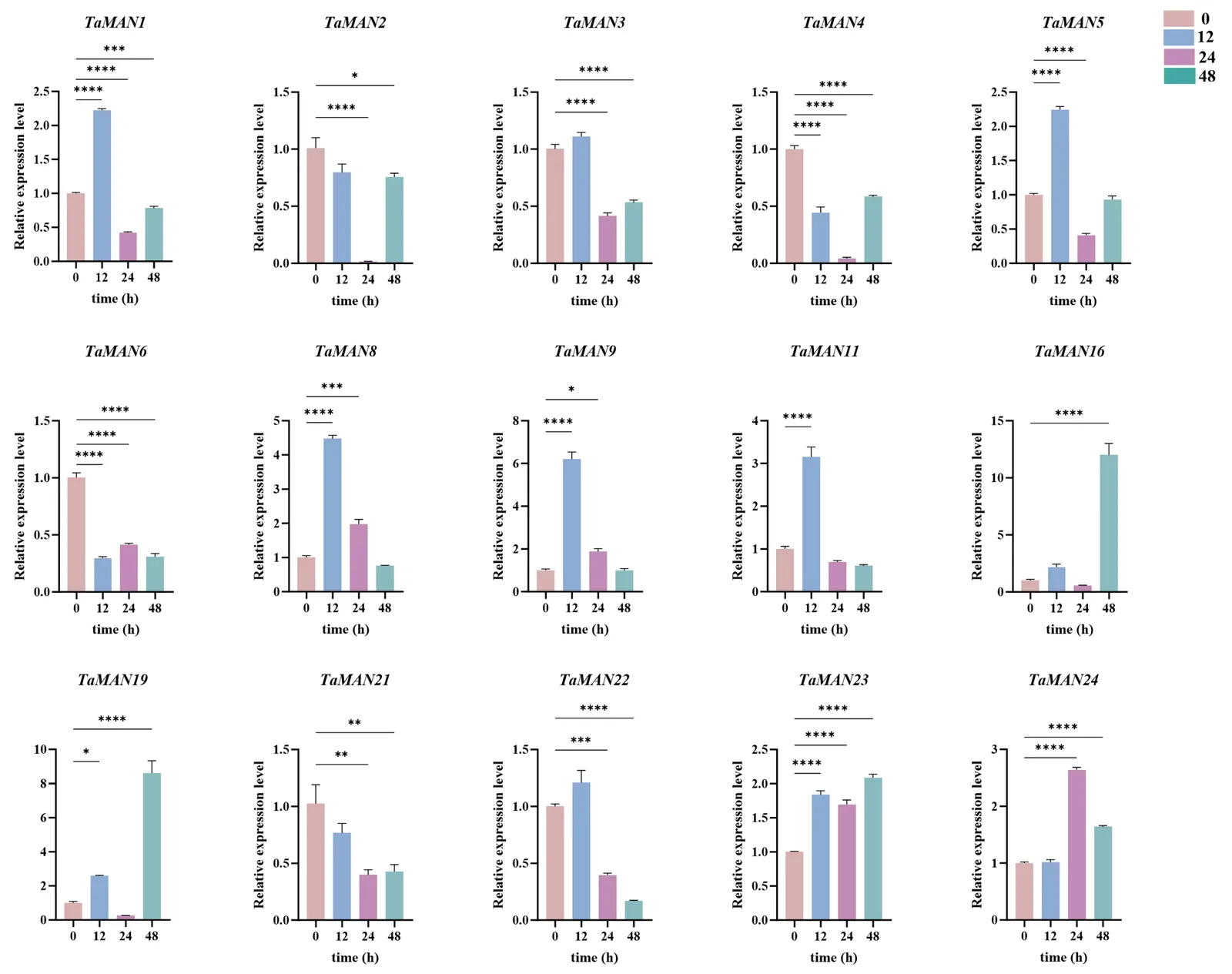
Gene expression patterns of nine *TaMAN* members under powdery mildew stress (E09). *, **, ***, and **** indicate the significant correlations at the levels of *p* < 0.05, *p* < 0.01, *p* < 0.001, and *p* < 0.0001 respectively.

To further verify the expression responses of *TaMAN* genes to pathogen stress, the relative expression levels of 15 *TaMAN* genes in leaves at 0, 12, 24 and 48 h after powdery mildew inoculation were determined by RT-qPCR (Figure 10, Table S7). The examined members exhibited three distinct response patterns. The first group, comprising *TaMAN1*, *TaMAN5*, *TaMAN8*, *TaMAN9* and *TaMAN11*, showed an early rapid-induction pattern: these genes were significantly induced at 12 h post-inoculation (hpi), reaching approximately 2.2- to 6.2-fold of the control (*TaMAN9* showed the largest induction, about 6.2-fold), and then gradually returned to near-control levels. The second group, comprising *TaMAN16* and *TaMAN19*, displayed a late strong-induction pattern: their expression changed little at the early stages but was strongly induced at 48 hpi, reaching approximately 11.5- and 8.7-fold of the control, respectively, representing the largest induction amplitudes among all examined members. The third group showed sustained or mid-stage responses: *TaMAN23* was continuously up-regulated from 12 to 48 hpi (approximately 1.7- to 2.1-fold), and *TaMAN24* peaked at 24 hpi (approximately 2.6-fold). In contrast, *TaMAN2*, *TaMAN3*, *TaMAN4*, *TaMAN6*, *TaMAN21* and *TaMAN22* were down-regulated to varying degrees after inoculation. These results indicate that *TaMAN* family members play differential roles at different stages of powdery mildew infection: *TaMAN1*, *TaMAN5*, *TaMAN8*, *TaMAN9* and *TaMAN11* are likely involved mainly in early defense responses, whereas *TaMAN16* and *TaMAN19* may function at later infection stages (haustorium formation and hyphal expansion). Together with the public-database expression analysis, in which *TaMAN1*, *TaMAN5* and *TaMAN23* were induced by powdery mildew (Figure 7D), *TaMAN1*, *TaMAN5*, *TaMAN8*, *TaMAN9*, *TaMAN16*, *TaMAN19*, *TaMAN23* and *TaMAN24* can be regarded as priority candidate genes for the wheat response to powdery mildew infection.

## 4. Discussion

### 4.1. Expansion and Evolution of the TaMAN Family in Allohexaploid Wheat

The 24 *TaMAN* genes identified in this study substantially outnumber the *MAN* complements reported for *Arabidopsis*, tomato, soybean and *Brachypodium* [28,40,62]. This expansion is consistent with the allohexaploid origin of bread wheat, which arose through two successive hybridization and polyploidization events involving three diploid progenitors [1,63]. Most *TaMAN* members were retained as A/B/D homoeologous copies distributed across the three subgenomes, mirroring the genome-wide pattern of homoeolog retention and balanced triad expression described in the transcriptional landscape of polyploid wheat [42]. Intraspecific synteny analysis indicated that segmental (whole-genome) duplication, rather than tandem duplication, was the principal driver of *TaMAN* family expansion. Interspecific collinearity further showed that *TaMAN* genes share far more orthologous pairs with rice and maize than with *Arabidopsis*, in line with the closer evolutionary relationships among *Poaceae* species, and several collinear blocks could be traced back to the diploid progenitors *Triticum urartu* and *Aegilops tauschii*, supporting an ancient origin of the *MAN* complement predating wheat polyploidization.

### 4.2. Conserved Protein Architecture and Cell Wall Targeting of TaMANs

Phylogenetic reconstruction grouped the TaMAN proteins into three clades together with functionally characterized MANs from *Arabidopsis*, rice and maize, and members of the same clade shared highly similar exon–intron organization and motif composition, supporting the robustness of the classification and the conserved evolutionary trajectory of the family [24,25]. The two catalytic glutamate residues that define the retaining double-displacement mechanism of GH5 enzymes were conserved in nearly all *TaMANs* [23], suggesting that the encoded proteins are bona fide mannan-cleaving hydrolases. Most TaMANs carry N-terminal signal peptides and were predicted to be secreted to the cell wall/apoplast [64], which is consistent with the extracellular location of their mannan substrates in the wall matrix [13,15] and with the experimentally determined cell wall localization of *MirMAN* [35]. The presence of a minority of TaMANs lacking obvious signal peptides suggests that some members may act intracellularly or have acquired distinct targeting routes during evolution.

### 4.3. Cis-Regulatory Architecture Links TaMAN Transcription to Hormone and Stress Signaling

Analysis of the 2.0-kb promoter regions revealed that *TaMAN* genes are enriched in hormone- and stress-responsive *cis*-acting elements, including ABA-responsive elements (ABRE), MeJA-responsive motifs, MYB-binding sites involved in drought inducibility (MBS), anaerobic-responsive elements (ARE), low-temperature-responsive elements (LTR) and defense/stress-associated TC-rich repeats. This cis-regulatory landscape indicates that *TaMAN* transcription is integrated into ABA- and jasmonate-mediated stress signaling networks [65], and is reminiscent of the strict hormonal control of *MAN* expression by gibberellin, ABA and ethylene documented during seed germination in tomato, lettuce and *Arabidopsis* [27,29]. The differential distribution of these elements among *TaMAN* members and clades further provides a plausible regulatory basis for their divergent stress-responsive expression patterns.

### 4.4. TaMAN Genes in the Salt-Stress Response

Public expression data and our own salt-stress RNA-seq of Dekang 961 converged on a subset of *TaMAN* genes, most prominently *TaMAN1*, *TaMAN5* and *TaMAN19,* which were strongly and sustainably induced by salinity, and the RT-qPCR profiles closely matched the RNA-seq trends, confirming the reliability of the transcriptomic data. How might a cell wall hydrolase contribute to salt tolerance? Mechanistic clues are provided by the heterologous expression of *MirMAN* in *Arabidopsis*, which improved survival under high salinity by enhancing antioxidant enzyme activities, reducing reactive oxygen species accumulation, increasing soluble sugar content and promoting primary and lateral root development [35]. Mannan hydrolysis releases mannose-containing oligosaccharides and free sugars that may contribute to osmotic adjustment, while wall loosening could facilitate the root growth plasticity required for salt avoidance [15,35]. In addition, *AtMAN3* confers cadmium tolerance through glutathione-dependent redox regulation [34], and the capacity to maintain redox homeostasis is a well-established determinant of salt tolerance in wheat [7,66]. Consistent with a broad involvement of wall metabolism, our transcriptome time series showed that cell wall organization and carbohydrate metabolic processes were prominently remodeled upon salt treatment, in agreement with previous wheat salinity transcriptomes [67,68]. Together, these data support the view that MAN-mediated wall remodeling is an integral, and previously underappreciated, component of the wheat salt response. Together, these data support the view that MAN-mediated wall remodeling is an integral, and previously underappreciated, component of the wheat salt response. More specifically, TaMAN-mediated mannan turnover may contribute to salt tolerance through at least three non-exclusive mechanisms: (i) osmotic adjustment—hydrolysis of wall mannans releases mannose and mannan-derived oligosaccharides that can serve as compatible solutes, lowering cellular osmotic potential and facilitating water retention under saline conditions; (ii) cell wall loosening and root growth plasticity—localized wall remodeling by secreted TaMANs may loosen the cell wall in elongation zones, enabling continued root growth through saline soil layers and thereby promoting salt avoidance; and (iii) redox homeostasis—given that *AtMAN3* confers cadmium tolerance through glutathione-dependent redox regulation [34], *TaMAN* members may similarly participate in maintaining cellular redox balance under salt-induced oxidative stress. The enrichment of ABRE and MeJA-responsive *cis*-acting elements in *TaMAN* promoters further suggests that ABA- and jasmonate-mediated signaling pathways orchestrate *TaMAN* expression during the salt stress response, integrating wall remodeling with hormonal stress signaling.

### 4.5. TaMAN Genes in the Wheat–Powdery Mildew Interaction

The cell wall is the first battlefield between wheat and powdery mildew: to establish feeding structures, *Bgt* must breach the epidermal wall, and the deposition of wall appositions (papillae) at penetration sites is a hallmark of basal and race-specific resistance [38,39]. Several *TaMAN* genes, most prominently *TaMAN5* and *TaMAN19*, were rapidly induced following *Bgt* inoculation in the present study. Two non-exclusive interpretations can be considered. On the one hand, pathogen-induced wall remodeling may constitute an active host defense strategy, because hydrolysis of wall polysaccharides can release oligosaccharide fragments that act as damage-associated molecular patterns to amplify immune signaling, as demonstrated for oligogalacturonides released by fungal endopolygalacturonases [69] and proposed for mannan-derived oligosaccharides generated by PtrMAN6 in poplar [17]. In this context it is noteworthy that other wall-associated enzymes and proteins, such as germin-like proteins and the isoprenylated HIPP1, are established components of wheat powdery mildew resistance [70,71]. On the other hand, excessive wall loosening could theoretically facilitate pathogen penetration, such that individual *TaMANs* might act as either resistance or susceptibility factors; their net contribution must therefore be resolved genetically, for example by virus-induced gene silencing or stable transformation. Regardless of the direction of the effect, the rapid and specific transcriptional activation of *TaMAN5* identifies it as a candidate effector of the wheat–*Bgt* interaction that merits functional validation.

### 4.6. TaMAN5 as a Dual-Stress Candidate Gene and Perspectives for Breeding

Integrating all lines of evidence, *TaMAN5* emerged as the most compelling candidate of the family: it was strongly induced by both salt stress and Bgt infection, its promoter harbors multiple stress- and hormone-responsive *cis*-acting elements, and its encoded protein is predicted to be secreted into the apoplast, the compartment in which both wall remodeling and the wheat–powdery mildew confrontation take place. Its close homologs in other species are linked to wall loosening and stress adaptation [17,34,35], further supporting functional conservation. From a translational perspective, mining natural allelic variation in *TaMAN5* and other stress-responsive *TaMANs* across wheat germplasm, followed by the development of functional molecular markers, could provide immediately usable tools for marker-assisted selection of salt-tolerant and powdery mildew-resistant wheat, complementing the successful deployment of cloned resistance genes such as *Pm21* and *Pm61* in breeding programs [72,73,74].

### 4.7. Limitations and Outlook

This study is based primarily on genome-wide bioinformatic prediction and transcript-level evidence. Although the concordance between public expression data, RNA-seq and RT-qPCR strengthens the reliability of the identified expression patterns, the enzymatic activity, exact subcellular localization and biological functions of TaMAN proteins, and TaMAN5, TaMAN19 in particular, remain to be verified experimentally, for example through heterologous expression and activity assays, subcellular localization in *Nicotiana benthamiana*, and gain- and loss-of-function analyses in wheat. Such experiments will clarify whether MAN-mediated mannan turnover contributes to stress tolerance through osmotic adjustment, redox homeostasis, wall-derived signaling, or a combination of these mechanisms, and will ultimately determine the value of *TaMAN* genes as targets for the genetic improvement of wheat stress resilience.

## 5. Conclusions

This study presents the first genome-wide systematic dissection of the endo-β-1,4-mannanase (*TaMAN*) gene family in common wheat. A total of 24 members were identified and divided into three groups; family expansion was dominated by whole-genome duplication and supplemented by tandem duplication, and the members were integrally retained during polyploidization and domestication. The promoter regions are enriched in hormone- and stress-responsive elements. Expression analyses combined with RT-qPCR identified pathogen stress candidate genes such as *TaMAN1*, *TaMAN5*, *TaMAN8*, *TaMAN9*, *TaMAN16*, *TaMAN19*, *TaMAN23* and *TaMAN24*, as well as early rapid salt-responsive genes such as *TaMAN3*, *TaMAN4* and *TaMAN19*-*TaMAN22*. These results may provide a systematic framework for dissecting the functions of *TaMAN* genes in wheat responses to biotic and abiotic stresses and offer valuable candidate gene resources for disease-resistant and salt-tolerant wheat molecular breeding.

## Figures and Tables

**Table 1:** Physicochemical properties and predicted subcellular localization of TaMAN proteins.

Gene Name	AA (aa)	Mw (Da)	pI	Instability Index	Aliphatic Index	GRAVY	Subcellular Localization
*TaMAN1*	425	45,875.56	9.3	26.66	72.4	−0.288	Extracellular
*TaMAN2*	444	49,881.6	5.8	41.87	84.37	−0.193	Endoplasmic reticulum
*TaMAN3*	425	45,713.34	9.11	28.84	71.48	−0.276	Extracellular
*TaMAN4*	444	49,817.52	5.93	42.06	83.92	−0.184	Endoplasmic reticulum
*TaMAN5*	425	45,748.33	9.11	25.64	71.72	−0.288	Extracellular
*TaMAN6*	445	49,928.51	5.74	41.69	84.83	−0.179	Endoplasmic reticulum
*TaMAN7*	471	51,862.2	5.59	29.16	78.13	−0.201	Extracellular, Lysosome/Vacuole
*TaMAN8*	420	46,816.79	4.61	38.35	80.19	−0.143	Extracellular, Lysosome/Vacuole
*TaMAN9*	420	46,977.9	4.54	39.88	79.95	−0.153	Lysosome/Vacuole
*TaMAN10*	472	52,085.71	5.97	30.68	80.89	−0.176	Extracellular
*TaMAN11*	386	43,207.28	4.47	37.47	77.15	−0.226	Cytoplasm
*TaMAN12*	475	52,343	6.02	30.46	80.78	−0.16	Endoplasmic reticulum
*TaMAN13*	426	46,404.37	7.13	38.25	74.04	−0.222	Extracellular, Lysosome/Vacuole
*TaMAN14*	424	47,091.3	6.98	40.17	73.18	−0.265	Extracellular, Lysosome/Vacuole
*TaMAN15*	413	45,879.12	8.74	43.97	68.26	−0.229	Extracellular, Lysosome/Vacuole
*TaMAN16*	427	46,680.68	8.79	42.15	69.74	−0.259	Extracellular, Lysosome/Vacuole
*TaMAN17*	402	44,383.41	6.03	35.93	76.67	−0.153	Extracellular, Lysosome/Vacuole
*TaMAN18*	413	45,643.84	9	48.49	67.14	−0.248	Extracellular
*TaMAN19*	426	46,590.65	8.56	37.81	68.29	−0.277	Extracellular, Lysosome/Vacuole
*TaMAN20*	420	46,322.41	7.02	39.51	73.4	−0.243	Extracellular, Lysosome/Vacuole
*TaMAN21*	413	45,652.7	7.72	48.54	69.47	−0.235	Extracellular
*TaMAN22*	397	44,637.38	5.1	42.13	87.98	−0.28	Cytoplasm
*TaMAN23*	437	49,266.17	6.83	41.13	88.63	−0.192	Vacuole
*TaMAN24*	429	48,456.29	8.61	43.38	87.09	−0.22	Vacuole

Abbreviations: AA (aa), Number of Amino Acid; MW (Da), Molecular Weight (Daltons); pI, Theoretical Isoelectronic Point; GRAVY, Grand Average of Hydropathicity.

## Data Availability

The original contributions presented in this study are included in the article. Further inquiries can be directed to the first author.

## Supplementary Materials

The following supporting information can be downloaded at: https://www.mdpi.com/article/10.3390/biology15171476/s1, Table S1: Protein information of *TaMAN* genes identified in this study; Table S2: Accenssion numbers of *AtMANs*, *OsMANs*, *ZmMANs* and *TaMANs*; Table S3: List of interspecific collinear gene pairs identified between *Triticum aestivum* (*TaMAN* members) and six plant species; Table S4: Characteristics of *cis*-acting regulatory elements in the promoter region of the identified *TaMAN* genes; Table S5: Clustering results of time-series data; Table S6: FPKM values of *TaMAN* gene family members across different stages and treatments; Table S7: Primer sequences used for *TaMAN* genes.

## Abbreviations

The following abbreviations are used in this manuscript:

RT-qPCR	Reverse Transcription Quantitative Polymerase Chain Reaction
GRAVY	Grand Average of Hydropathy
pI	isoelectric points
ABA	abscisic acid
MBS	Matrix Binding Site
LTR	Long Terminal Repeat
MRE	Metal Response Element
*Bgt*	*Blumeria graminis* f. sp. *tritici*
